# Causality research based on phase space reconstruction

**DOI:** 10.1371/journal.pone.0313990

**Published:** 2024-11-22

**Authors:** Lei Hu, Zhuoma Sunu, Hongke She, Binghuai Fan, Jingru Ma, Chaojiu Da

**Affiliations:** 1 School of Mathematics and Computer Science Institute, Northwest Minzu University, Lanzhou, China; 2 Institute of Applied Mathematics and Astronomical Calendar, Northwest Minzu University, Lanzhou, China; ICIMOD: International Centre for Integrated Mountain Development, NEPAL

## Abstract

Based on phase space reconstruction theory, the root mean square error is used as a quantitative criterion for identifying the appropriate embedding dimension and time step and selecting the optimal configuration for these factors. The phase space is then reconstructed, and the convergent cross-mapping algorithm is applied to analyse the causality between time series. The causality among the variables in the Lorenz equation is first discussed, and the response of this causality to the integration step of numerical solutions to the Lorenz equation is analyzed. We conclude that changes in the integration step do not alter the causality but will affect its strength. Variables *X* and *Y* drive each other, whereas variable *Z* drives variables *X* and *Y* in a unidirectional manner. Second, meteorological data from 1948–2022 are used to analyse the effect of the Southern Hemisphere annular mode on the East Asian summer monsoon index and surface air temperature driving capacity. From a dynamic perspective, it is concluded that the Southern Hemisphere annular mode is the driving factor affecting the East Asian summer monsoon index and surface air temperature. Based on ideal test results and the observation data, the collaborative selection of the embedding dimension and time step is more reliable in terms of determining causality. This provides the ability to determine causality between climate indices and theoretically guarantees the selection of climate predictors.

## 1. Introduction

Atmospheric motion is an extremely challenging topic because of its multiscale nature, complex dynamics, and thermodynamic effects. In the end of the 19th century and beginning of the 20th century, Bjerknes established a system of atmospheric dynamics equations, providing a milestone in human understanding of atmospheric motion [1]. The highly nonlinear nature and complexity of atmospheric dynamics means that analytical solution to these equations could not be obtained, leaving only numerical solutions. There are several models for predicting atmospheric motion, including dynamic analysis models [2,3], statistical models [4], and dynamic-statistical models [5–7]. The key to the establishment of statistical models is the selection of predictors, which essentially describe the driving causality between various elements of atmospheric motion [8]. Therefore, the study of causality is an important issue in atmospheric science. Causality is applicable in various disciplines [9–11], reflecting the relationships that stimulate and are stimulated between things. These relationships can reveal the inherent connections among quantities in the process of development and change. Against the backdrop of global warming, the inference of causality in the climate system can be used to project the risks associated with climate change, particularly in attributing the cause of extreme weather events.

Since the 1970s, attribution research has made significant progress in both theory and application based on a considerable amount of observational data [12–15]. In atmospheric science, meteorological research institutions in various countries now have access to large amounts of observation and model output data following innovations in observation technology, improved storage conditions, faster calculation methods, and easier means of communication. The mining of information from these data and the extraction of reliable predictors are urgent scientific problems. Granger first proposed a causality test method for strongly coupled systems in 1969 [16]. The premise of this causal test is that the time series must be stationary; otherwise, false attribution conclusions may appear. Since then, many scholars have proposed various attribution theories and methods [17,18], such as causal network learning [19,20]. In 1981, Takens proposed the theory of phase space reconstruction [12], which aims to reconstruct a phase space that is diffeomorphic to the solution space of the original differential equations. This method employs the time series of a solution component from a system of differential equations, selects the appropriate embedding dimension and time step, and uses this time series as a predictor for other solution components of the differential equation group. Based on Takens’ theory, a lot of attribution research has been done. Some scholars have focused on parameter determination; for example, Fraser and Swinney proposed the mutual information method [21], and Li et al. discussed the theory of determining the attractor dimension by reconstructing the phase space with a one-dimensional time series [22]. In terms of the application of attribution theory, Feng et al. developed the dynamical correlation factor exponent, which enables us to study the dynamic structural features of the climate system [23]. Takens’ theory is also widely used in biological mathematics, mainly in the construction of biological models. Sugihara et al. introduced the technique of convergent cross-mapping (CCM), which complements Granger’s causality [24]. This causality in weakly coupled nonlinear systems can be tested, allowing reliable predictors to be obtained and producing more accurate prediction conclusions [8,25–27].

Over the past few years, significant progress has been made in using dynamic causality to analyze drivers of the climate system. Wang et al. used the CCM algorithm to investigate the causal relationship between Northern Hemisphere annular modes and winter surface air temperature (SAT) over Northeast Asian [28,29]. Tsonis et al. using CCM to explore the dynamic relationship between cosmic rays and global temperature [30], while Chen et al. used the CCM algorithm to analyze meteorological drivers affecting PM2.5 concentrations in China [31]. These studies have contributed to our understanding of the complex dynamics of climate systems.

In the previous CCM causal detection process, the embedded dimension is often determined separately, and the time delay is usually obtained by experience, without considering the common influence of parameters in detail [28–31]. In this paper, a collaborative parameter determination method is proposed to improve the testing ability of causality strength. The remainder of the article is structured in the following manner. Section 2 contains Basic theory and CCM algorithm. Section 3 contains Parameter selection method in ideal type test. Section 4 contains the driving capability of the Southern Hemisphere annular mode (*SAM*) index on East Asian summer *SAT* and the East Asian summer monsoon index (*EASMI*) will be explored. Section 5 contains conclusion and prospect.

## 2. Methods and data

### 2.1. Takens’ theorem [12]

Let *M* be a compact *d*-dimensional manifold. Given a pair of transformations (*φ*,*y*), where *φ*: *M*→*M* is a smooth diffeomorphism and *y*: *M*→*R* is a smooth function, we define a mapping as follows:

$$F_{\left(\varphi ,y\right)}(x)=(y(x),y(\varphi (x)),\cdots ,y(\varphi^{2d}(x)))$$ (1)

This is an embedding map from *M* to the space *R*^2*d*+1^. Moreover, the smoothness of the mapping is guaranteed by the fact that it has at least a second-order continuous derivative, as stated in the theorem.

Takens’ theorem establishes the minimum dimension required to guarantee the existence of a diffeomorphism. In many cases, it is unnecessary to extend the dynamics to such high dimensions. However, accurately determining the minimum dimension in actual data experiments can be challenging, highlighting the importance of identifying the optimal embedding dimension for the success of the experiment.

### 2.2. Convergent cross-mapping algorithm [24]

Consider two time series *X* and *Y* of length *L* from the same system. We obtain

$$X(t)=\{x_{1},x_{2},\cdots ,x_{L}\}$$ (2)

$$Y(t)=\{y_{1},y_{2},\cdots ,y_{L}\}$$ (3)

where $x_{i}=x(t_{i})(1\leq i\leq L,i\in N)$, and similarly for *y*_*i*_, represent the values of the time series *X*, *Y* at time *t*_*i*_. Suppose that the embedding dimension is *E* (according to Takens’ theory, *E* must be greater than 2*d*+1) and the time step is *τ*, where both *E* and *τ* are natural numbers. To discuss the causality between *X* and *Y*, we define the embedding vectors of variables *X* and *Y* at time *t*_*j*_ as *M*_*X*_(*t*_*j*_) and *M*_*Y*_(*t*_*j*_), respectively. The sets of embedding vectors of *M*_*X*_(*t*_*j*_) and *M*_*Y*_(*t*_*j*_) constitute the phase spaces *M*_*X*_ and *M*_*Y*_, respectively. For example, consider *X*:

$$M_{X}(t_{j})=(x_{j-(E-1)\tau },x_{j-(E-2)\tau },\ldots ,x_{j-\tau },x_{j})$$ (4)

$$M_{X}=\{M_{X}(t_{j})|(E-1)\tau +1\leq j\leq L,j\in N\}$$ (5)

Where {*j*}⊂{*i*}. We define $\hat{X}(t)|M_{Y}$ as the prediction of the time series *X*(*t*) by applying the cross-mapping through the phase space *M*_*Y*_, and similarly for $\hat{Y}(t)|M_{X}$.

At time *t*_*j*_, we search for the *E*+1 nearest neighbours of *M*_*Y*_(*t*_*j*_) in the embedded vector set *M*_*Y*_, sorted in ascending order of distance as *M*_*Y*_(*t*_*j*1_), *M*_*Y*_(*t*_*j*2_), …, *M*_*Y*_(*t*_*j*(*E*+1)_), where $\left\{t_{jk}(1\leq k\leq E+1)\}\subset \{t_{j}((E-1)\tau +1\leq j\leq L)\right\}$. Assuming that the mapping of these *E*+1 nearest neighbours on *M*_*X*_ is*M*_*X*_(*t*_*j*1_), *M*_*X*_(*t*_*j*2_), …, *M*_*X*_(*t*_*j*(*E*+1_), we can obtain the predicted value of $\hat{X}(t_{j})|M_{Y}$ at time *t*_*j*_ by the cross-mapping, which is given by:

$$\hat{X}(t_{j})|M_{Y}=\sum_{k=1}^{E+1}w_{jk}x_{jk}$$ (6)

Where *w*_*jk*_ is the distance weight of vector *M*_*Y*_ in the set *M*_*Y*_(*t*_*j*_) to the kth nearest neighbour *M*_*Y*_(*t*_*jk*_), i.e., $w_{jk}=u_{jk}/\sum_{k=1}^{E+1}u_{jk}$, $u_{jk}=e^{-\frac{d[M_{Y}(t_{j}),M_{Y}(t_{jk})]}{d[M_{Y}(t_{j}),M_{Y}(t_{j1})]}}$, and (.,.) is the Euclidean distance. Obviously,

$$u_{j(E+1)}\leq u_{jE}\leq u_{j(E-1)}\leq \ldots \leq u_{j1}=e^{-1}$$ (7)

### 2.3. Data

Data for monthly SAM index and EASMI data provided by Professor Li (http://lijianping.cn/dct/page/65577). The monthly SAT data at 1000 hPa are provided by the National Centers for Environmental Prediction, with a horizontal resolution of 2.5°×2.5° (https://www.psl. noaa.gov/data/gridded/data.ncep.reanalysis.html). The research area covers the East Asia region (4°N–53°N, 73°E–150°E), and the time span of our study ranges from January 1948 to December 2022. We consider the summer season to be the period from June to August.

## 3. Numerical results for Lorenz system

### 3.1. Data Solution for embedding dimension and time step

The Lorenz equation can be written as

$$\{\begin{array}{c} \dot{x}=\sigma (y-x)\\ \dot{y}=\rho x-y-xz\\ \dot{z}=xy-\beta z \end{array}$$ (8)

When *σ* = 10, $\rho =\frac{8}{3}$ and *β* = 28, chaos will occur and a chaotic attractor will be generated [32]. Taking the initial values as *x*(0) = 3, *y*(0) = 5 and *z*(0) = 9 and setting the integration interval and integration step to be [0,500] and Δ*t* = 0.1, respectively, we select the numerical solution of [350,500] as the test sequence, where *X* is the numerical solution of *x*, and *Y* and *Z* are obtained in a similar manner. The root mean square error (*RMSE*) is constructed as

$$R(E,\tau )=\sqrt{\frac{1}{L-(E-1)\tau }\sum_{j=(E-1)\tau +1}^{L}{\left[A(t_{j})-\hat{A}(t_{j})|M_{B}\right]}^{2}}$$ (9)

where *A* and *B* are any two of the time series *X*, *Y*, and Z. *A*(*t*_*j*_)is the numerical solution of variable *A* at time *t*_*j*_. When *E* and *τ* are given, $\hat{A}(t_{j})|M_{B}$ is the predicted value of variable *A* at time *t*_*j*_. The time step *τ* represents *τ* times the integration step, i.e., the sampling interval of *τ* Δ*t* time units. Eq (9) gives the *RMSE* as a function of *E* and *τ*.

**Fig 1:**
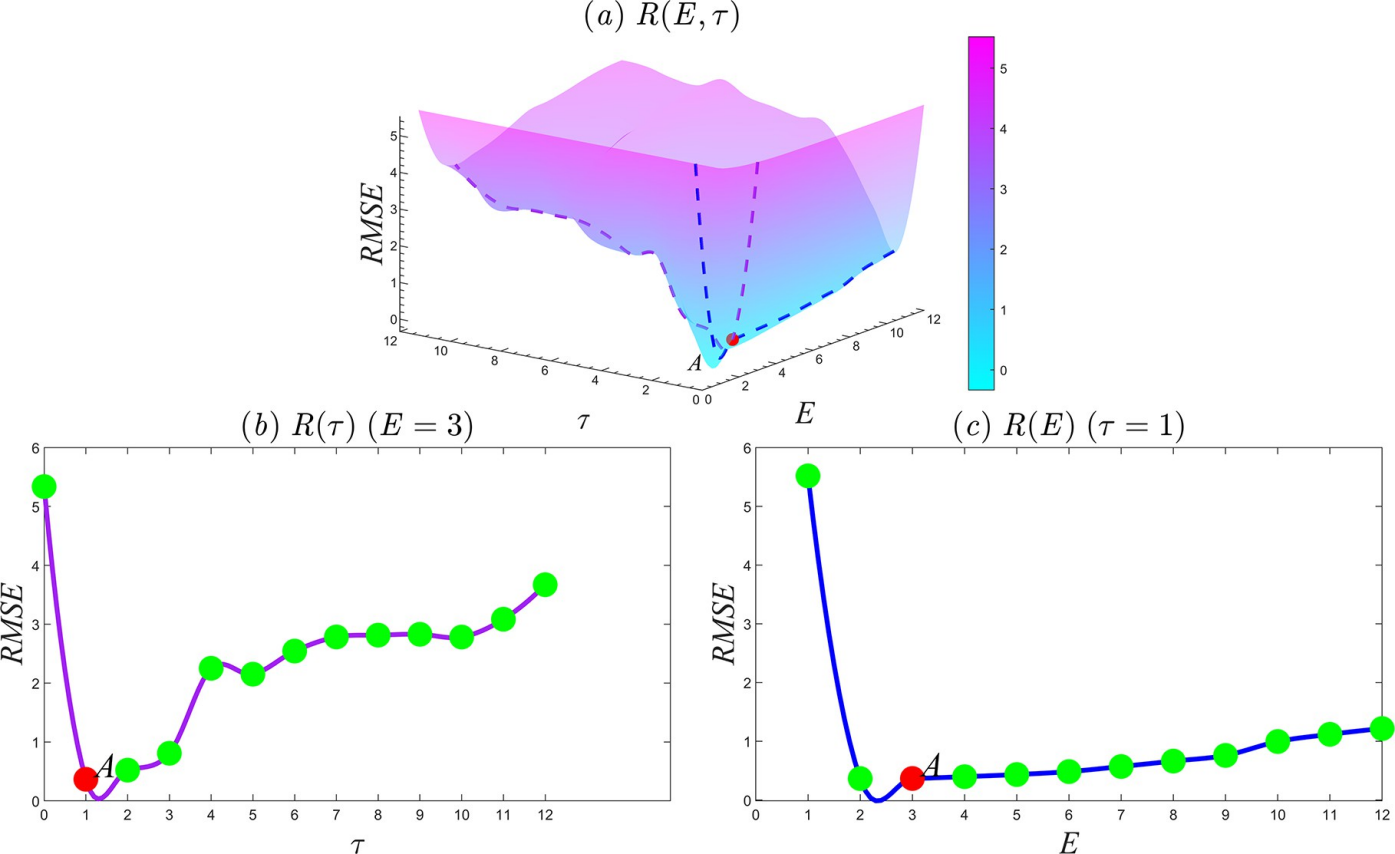
*RMSE* changes with the number of embedding dimensions and time steps. (a) *R*(*E*,*τ*), (*E*,*τ*)∈[1,12]×[0,12]; (b) *R*(*τ*), *E* = 3, *τ*∈[0,12]; (c) *R*(*E*), *τ* = 1, *E*∈[1,12].

Fig 1 shows the *RMSE* in the interval (*E*,*τ*)∈[1,12]×[0,12] by taking *A* as the time series *Y* and *B* as the time series *X*. To obtain a smooth image, cubic convolution interpolation has been performed based on lattice data, where Fig 1A is *R*(*E*,*τ*) and 1b and 1c show *R*(*τ*) (with E = 3) and *R*(*E*) (with *τ* = 1), respectively. When the embedding dimension *E* is greater than two, the correlation coefficients between the predicted values and the numerical solutions for each parameter configuration pass the 95% significance test. Fig 1A indicates that the smallest prediction error occurs when *E* = 3 and *τ* = 1, i.e., the red point *A* (because *E* and *τ* can only be positive integers). Moreover, when the embedding dimension *E* is given, the prediction error first decreases and then increases with increasing time step *τ*. When *τ* is fixed and the embedding dimension *E* is increased, the prediction error suddenly decreases and then slowly increases. That is, we again observe an initial decrease followed by an increase; Fig 1B and 1C exhibit similar trends. Based on these results, The following conclusions can be derived: (1) The reconstruction of the phase space of variable *X* provides geometrical information about variable *Y*; (2) When the time step exceeds a certain threshold, the prediction error tends to increase as the time step becomes larger, indicating that the reconstructed phase space cannot fully recover historical information. This suggests that more historical data may not necessarily provide more effective information, and could even introduce invalid information. **Therefore, the requirement for collaborative selection of the best parameters is that the parameters are positive integers; the prediction error is small and passes the 95% significance test.** Thus, for the following Lorenz equation test, we set *E* = 3 and *τ* = 1. This is consistent with the selection of parameters obtained by previous research methods [29].

In this paper, we consider collaborative parameter determination to improve the ability of causal detection. The simple algorithm is as follows:

**Table:** 

**Step 1**	Fix the maximum length of time series *X*, *Y*, and select the cross mapping under different *E* and *τ* in turn. Calculate the *RMSE* for different parameter pairs.
**Step 2**	The parameters are integers, the *RMSE* (Eq (9)) is minimum and the 95% significance test is used as the criterion to determine the parameters.
**Step 3**	According to the selected parameters, the correlation coefficients (Eq (10)) under different windows are calculated according to the different window lengths of the time series.
**Step 4**	Determine causality based on the degree of convergence of correlation coefficient with time length.
**Step 5**	Adjust the time series density and return to calculate steps 1–4.

### 3.2. Detection of causality

The correlation coefficient between the time series *A*(*t*_*j*_) *A*(*t*_*j*_) and $\hat{A}(t_{j})|M_{B}$ is given by

$$\rho (L)=\frac{\sum_{j=(E-1)\tau +1}^{L}\left[A(t_{j})-\bar{A}][\hat{A}(t_{j})|M_{B}-\bar{\hat{A}|M_{B}}\right]}{\sqrt{\sum_{j=(E-1)\tau +1}^{L}{\left[A(t_{j})-\bar{A}\right]}^{2}}\sqrt{\sum_{j=(E-1)\tau +1}^{L}{\left[\hat{A}(t_{j})|M_{B}-\bar{\hat{A}|M_{B}}\right]}^{2}}}$$ (10)

where $\bar{A}$ represents the mean value of *A*(*t*_*j*_) and $\bar{\hat{A}|M_{B}}$ represents the mean value of $\hat{A}(t_{j})|M_{B}$. If the limit converges, i.e.,

$$\underset{L\to \infty }{\lim}\rho (L)=u$$ (11)

then variable *A* is considered the driving factor of *B*, denoted by *A*↦*B*, showing that there is unidirectional causality between the two. The strength of causality is represented by the absolute value of the limit *u*, with stronger causality indicated by values closer to ±1. A lack of causality or weak causality is indicated by values further away from ±1. Convergence in this context means that *ρ*(*L*) approaches a certain value as *L* increases.

There are two methods for extending time series in numerical simulations of differential equations. One is to adjust the size of the integration step while maintaining the constant integration interval to increase the value of *L*. The other is to fix the integration step and increase the integration interval to make *L* larger. In observation data, the time series length can be extended by decreasing the observation interval or by collecting additional data.

**Fig 2:**
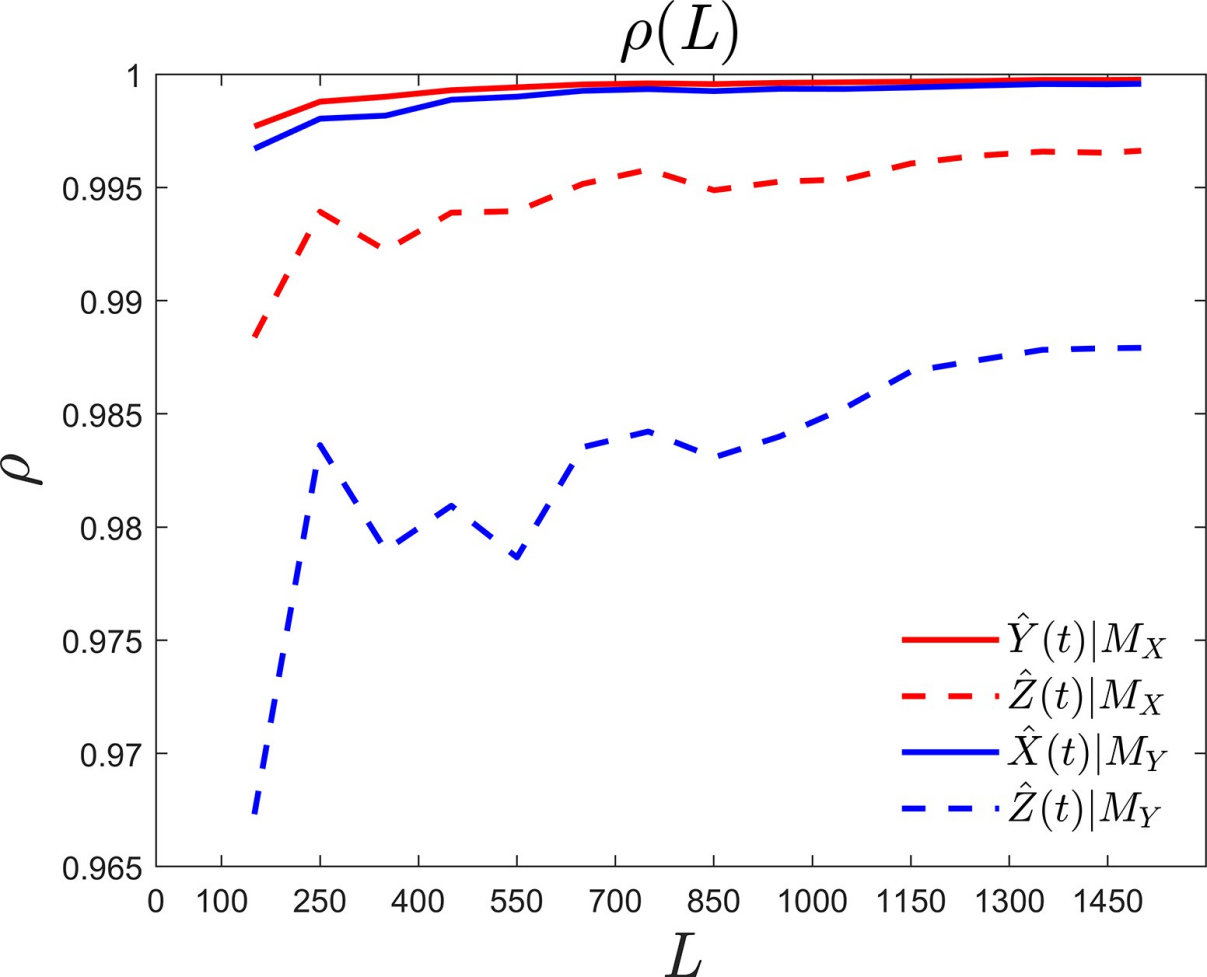
Causality curves between Lorenz variables.

Fig 2 displays the correlation coefficient curve given by Eq (10) in numerical simulations of the Lorenz Eq (8), where the abscissa represents the time series length, the ordinate *ρ* represents the correlation coefficient between the predicted value and the numerical solution. The predictions of variables *Y* and *Z* by the reconstructed phase space *M*_*X*_ are depicted by red solid lines and dashed lines, respectively. The predictions of variables *X* and *Z* by phase space *M*_*Y*_ are shown by solid and dashed blue lines, respectively. According to Takens’ theorem, the reconstructed phase space of variables *X* and *Y* is equivalent to the original phase space. However, for variable *Z*, the detection process is successful when *Z* is used as the predicted value, but fails when *Z* is used as the predicted field due to the rotational symmetry of the phase space reconstructed by *Z*. E. Deyle presented some forms of Takens’ theorem extension, but further research is still needed for univariate causal detection [33]. Thus, Fig 2 demonstrates the driving capability for detection success. These four correlation coefficient curves converge as the length *L* of the time series increases. The convergence values are all close to or above 0.99, indicating that *Y* and *Z* are the driving factors of *X*, and that *X* and *Z* are the driving factors of *Y*, with extremely strong causality, namely *Y*↦*X*, *Z*↦*X* and *X*↦*Y*, *Z*↦*Y*. However, the detection of *X*↦*Z*, *Y*↦*Z* fails due to the property of rotational symmetry in the reconstructed phase space of variable *Z* [33].

**Fig 3:**
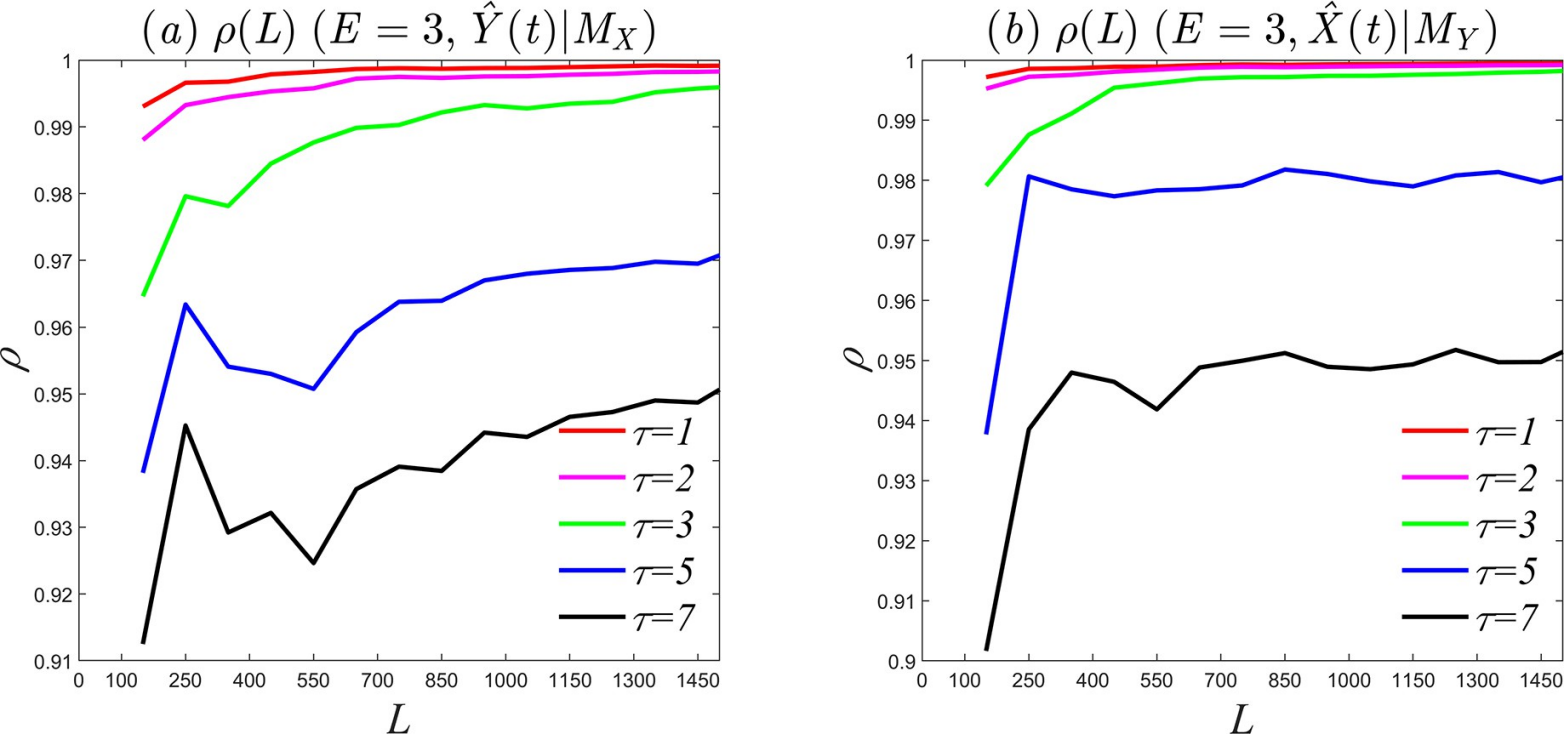
Intensity of causality. (a) Strength of the causal relationship between *Y* and *X*; (b) strength of the causal relationship between *X* and *Y*.

To assess the effect of the time step on causality, we set the embedding dimension to *E* = 3 and analyze causality in different time steps. Fig 3A shows the curve of the correlation coefficient between *Y* and $\hat{Y}(t)|M_{X}$ with different time steps. The results show that causality still exists as the time step increases, but the intensity of causality decreases. Fig 3B shows the causality between variables *X* and $\hat{X}(t)|M_{Y}$ with different time steps, which is similar to Fig 3A. Combined with Fig 1, it can be concluded that if the time step is too large, the prediction error may increase, the correlation will decrease, and the reconstructed phase space will not fully recover the system information. This will result in the loss of valid information and the inclusion of invalid information. However, based on Takens’ theorem, without considering the impact of computational cost, causality can still be detected through CCM by appropriately increasing the embedding dimension and time step. Thus, we have the following: (1) The CCM detection process is not random, i.e., appropriately increasing the embedding dimension and time step will not destroy causality, as shown by Fig 3; (2) To determine the strength of the causality, it is necessary to select the best embedding dimension and time step; (3) The study found that while the delay must fall within the system’s pseudo-period range of 0 to 1/4, increasing the delay appropriately does not alter the driving force, but rather its magnitude. Table 1 reveals a significant correlation between delay and data density. Importantly, increasing the delay appropriately when data density is higher does not undermine the test. In these cases, the reconstructed phase space remains diffeomorphic to the original system, with no loss of information due to excessive delay. This highlights the importance of considering the delay corresponding to different data densities when detecting causality in complex systems, and emphasizes the need to further explore the relationship between delay and data density.

**Table 1:** Forecast error with different integration steps.

Δ*t*	*E*	*τ*	*RMSE*
0.01	6	6	0.064
0.02	6	3	0.112
0.03	6	2	0.126
0.04	8	1	0.165
0.05	7	1	0.156
0.06	6	1	0.168
0.07	5	1	0.190
0.08	5	1	0.199
0.09	4	1	0.196
0.10	3	1	0.246

**Fig 4:**
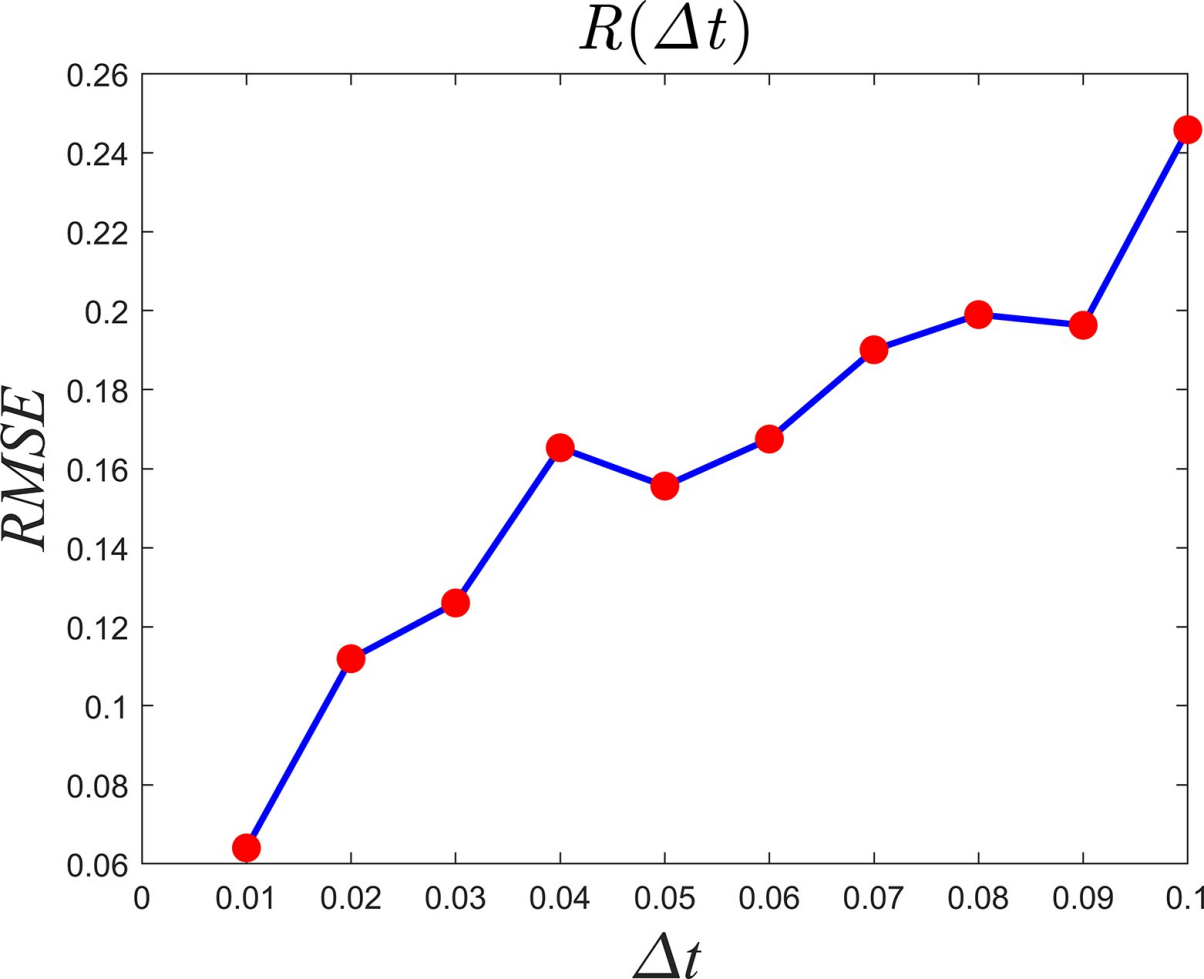
Causality curves between Lorenz variables.

Large amounts of data can be obtained from each national climate observation station across different observation periods, with the data density representing the different observation periods. Now, we theoretically discuss the impact of data density on the causality relationships in atmospheric observational data. The Lorenz equation is taken as the study object, with the initial conditions being *x*(0) = 3, *y*(0) = 5, and *z*(0) = 9, the integration interval being [0,500], and the integration step sizes successively set to 0.01, 0.02,…, 0.1. The numerical solution over the integration interval [350,500] is chosen as the test sequence. For example, when Δ*t* = 0.01, there are 15,000 numerical solutions for the test data. A smaller integration step gives a smoother time series and a greater data density. The optimal embedding dimension and time step are calculated from Eq (9). Fig 4 shows the prediction error of variable *X* on $\hat{X}(t)|M_{Y}$. As the integration step increases (i.e., the data density decreases), the prediction error increases, but the causality remains unchanged. This shows that a smaller integration step size and smoother data result in a smaller prediction error. Therefore, in detecting the causality of observation data, it is essential to select data that are as dense as possible.

**Fig 5:**
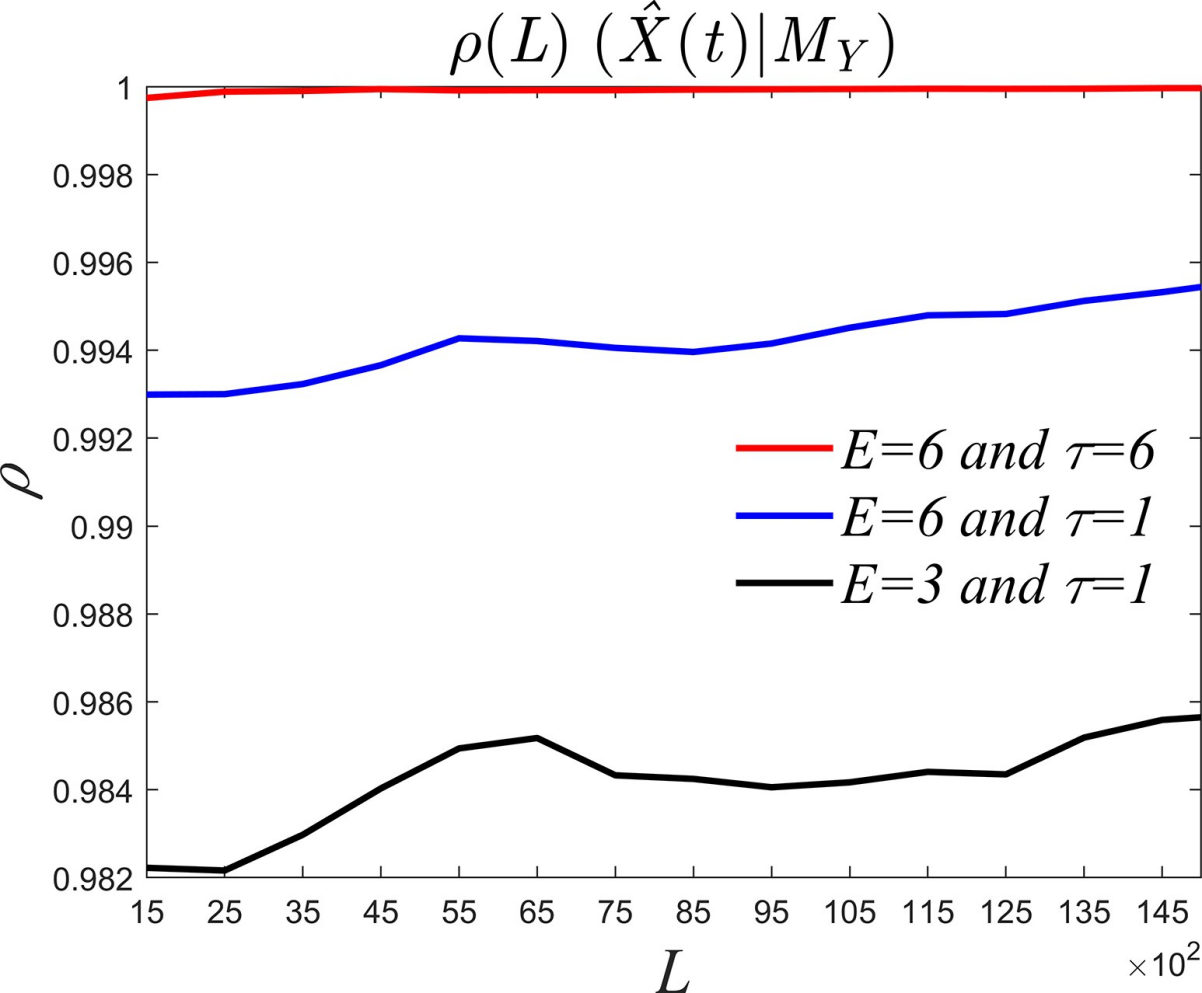
Convergence for different parameter configurations.

To compare the reliability and advantages of collaborative parameter determination, the initial field of the Lorenz Eq (8) remains unchanged while a numerical solution with an integration step of 0.01 and integration interval [350,500] is selected as the experimental sequence. Take the variables *X*, *Y* as an example. There are three schemes for parameter determination. One is *E* = 3 obtained according to Simplex Projection algorithm and *τ* = 1 obtained empirically [29]; the other is *E* = 6 and *τ* = 6 determined collaboratively in this paper; the third is to construct a control test and choose *E* = 6 and *τ* = 1 according to experience. As shown in Fig 5, when *E* = 6 and *τ* = 6, the convergence effect is the best with the largest convergence value, followed by the control test. Conversely, *E* = 3 and *τ* = 1 result in the worst convergence with the smallest convergence value. This is because when discrete data approaches continuous infinity, the selection of tau will affect the ability of the shadow manifold to recover information. If tau is too small and the dimension is too small, information overlap occurs, hence *E* and *τ* needs to be determined collaboratively under different density data. This also verifies the advantages of collaborative selection [12].

## 4. Causality detection of SAM and some summer climate indices in East Asia

### 4.1. Data analysis

The Southern Hemisphere annular mode is the dominant mode of variability in atmospheric circulation in the mid-to-high latitudes of the Southern Hemisphere [34]. However, the effects of *SAM* on climate are not limited to the Southern Hemisphere, and studies of the global climate often incorporate *SAM* [35,36]. Li et al. investigated the Northern Hemisphere climate by studying the *SAM* phase and climate index [37].

**Fig 6:**
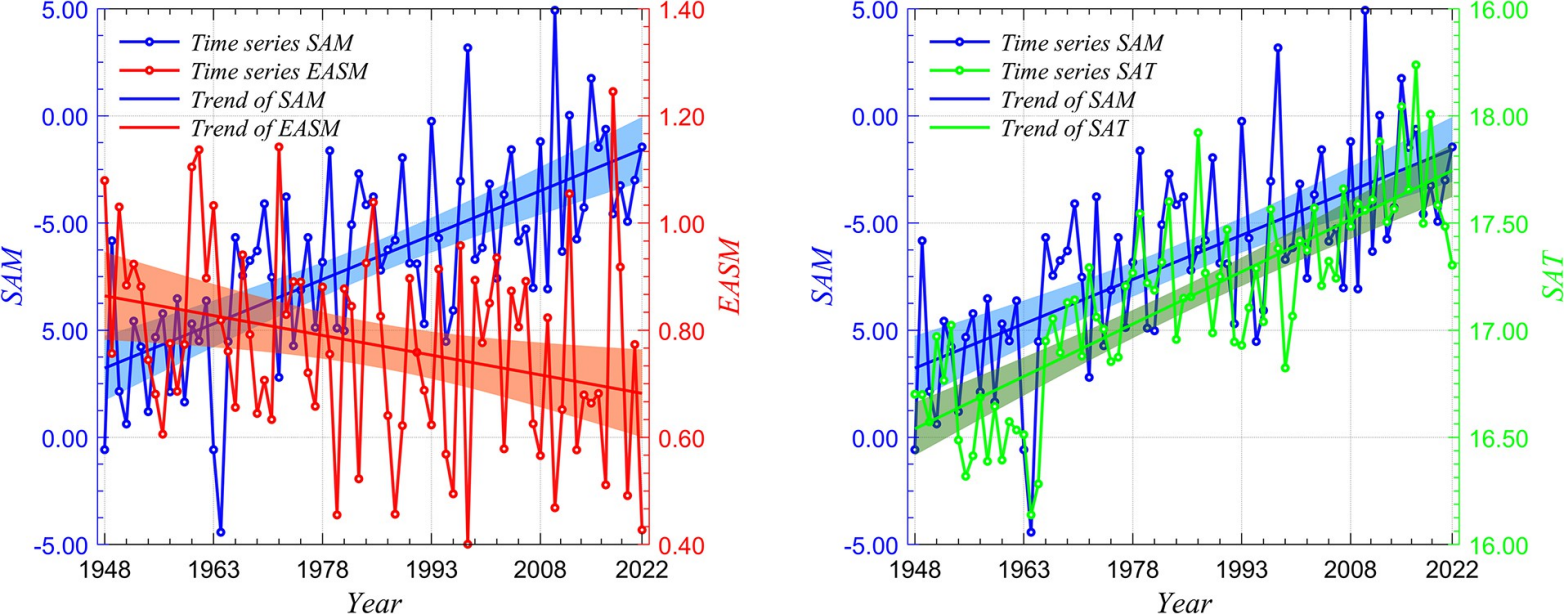
Season by season time series of *SAM*, *EASM* and 1000hPa *SAT* from 1948–2022 (blue: *SAM*; Orange: *EASM*; Green: *SAT*). (a)Time series and linear trend of *SAM* and *EASM* in summer; (b)Time series and linear trend of *SAM* and 1000hPa *SAT* in East Asian summer.

Although it is widely known that observed data inherently contain noise, this paper does not specifically focus on this aspect. However, in this study, the second-best parameter configuration was selected to minimize the impact of noise on the test results. In this section, we preliminarily analyze the correlation between summer *SAM* and *EASMI* and between summer *SAM* and the East Asian summer 1000*hPa SAT* from 1948–2022. Fig 6 shows the time series and the linear trend of each climate index from 1948–2022. The seasonal time series curves of *SAM* (solid line marked in blue) and *EASM* (solid line marked in orange) in summer from 1948–2022 are shown in Fig 6A. The correlation coefficient between variables is -0.36, and the correlation coefficient passes 95% significance test, indicating that the two have a good correlation. The regression analysis of *SAM* and *EASM* is carried out respectively, and it is found that *SAM* and *EASM* have obvious linear trend, and their linear trend coefficients are 0.67 and -0.29. Fig 6A The blue and orange bands show the confidence intervals with 95% significance for *SAM* and *EASM* regression analysis. Fig 6B shows the seasonal time series curves of summer *SAM* and East Asian summer 1000hPa *SAT* (solid line marked with green). There is also a significant correlation between *SAM* and *SAT*, with a coefficient value of 0.66. In addition, the linear trend coefficient of *SAT* is 0.80, and the green band is the regression analysis of *SAT*, passing the confidence interval at 95% significance level. The correlation coefficient between *SAM* and *SAT* was 0.66 from 1948–2022, and the correlation between *SAM* and *SAT* was negative from 1953–1963. In the mid-to-late 1970s, the correlation was positive again. There are obvious oscillations in local time periods. Traditional statistical analysis may yield different causality results in different time periods. In addition, correlation cannot establish causality in the physical sense. This section explores the causality between *SAM* and the East Asian summer climate based on Takens’ theory and the CCM algorithm. We also discuss the influence of different data densities on causality. Provide theoretical support for literature [37] as much as possible.

### 4.2. Causality between summer SAT and SAM in East Asia

**Fig 7:**
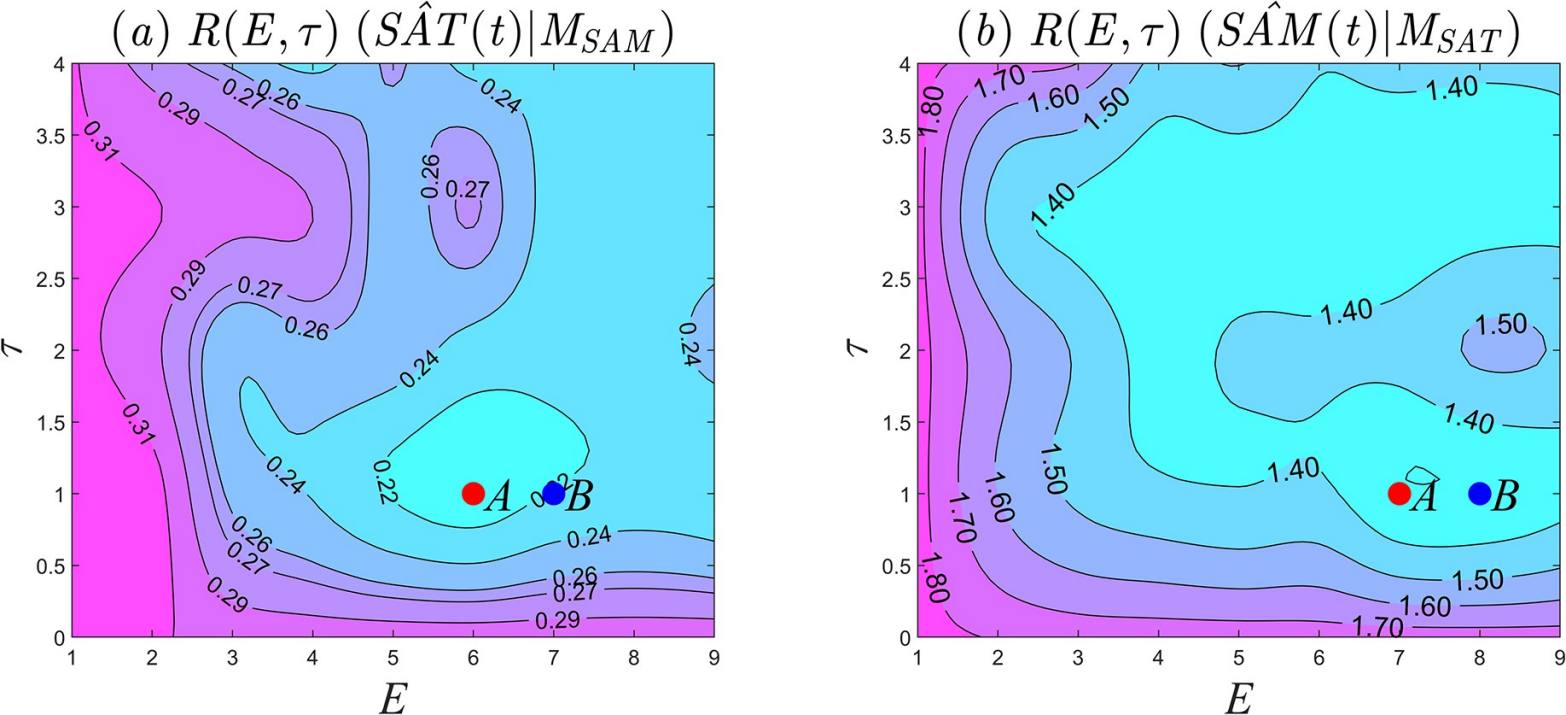
Determination of embedding dimension and time step. (a) *RMSE* of variable *SAT* under different parameters; (b) *RMSE* of variable *SAM* under different parameters.

To investigate the causality between summer *SAM* and *SAT* in East Asia, the optimal embedding dimension and time step for reconstructing the phase space are first determined, and the optimal parameter configuration is jointly selected. Fig 7 shows a contour map of *RMSE* given by Eq (9). For phase space reconstruction of the summer *SAM* time series, the optimal parameter configuration for minimizing the prediction error and passing the 95% significance test is *E* = 6 and *τ* = 1, as demonstrated by the red point *A* in Fig 7A. However, for phase space reconstruction of the *SAT* season-by-season time series, the parameter configuration that produces the smallest prediction error while still passing the 95% significance test is *E* = 7 and *τ* = 1, as shown by the red point *A* in Fig 7B. As control experiments, the parameter configurations with the next-smallest prediction errors are indicated by the blue points *B* in Fig 7A (*E* = 7, *τ* = 1) and 7b (*E* = 8, *τ* = 1).

**Fig 8:**
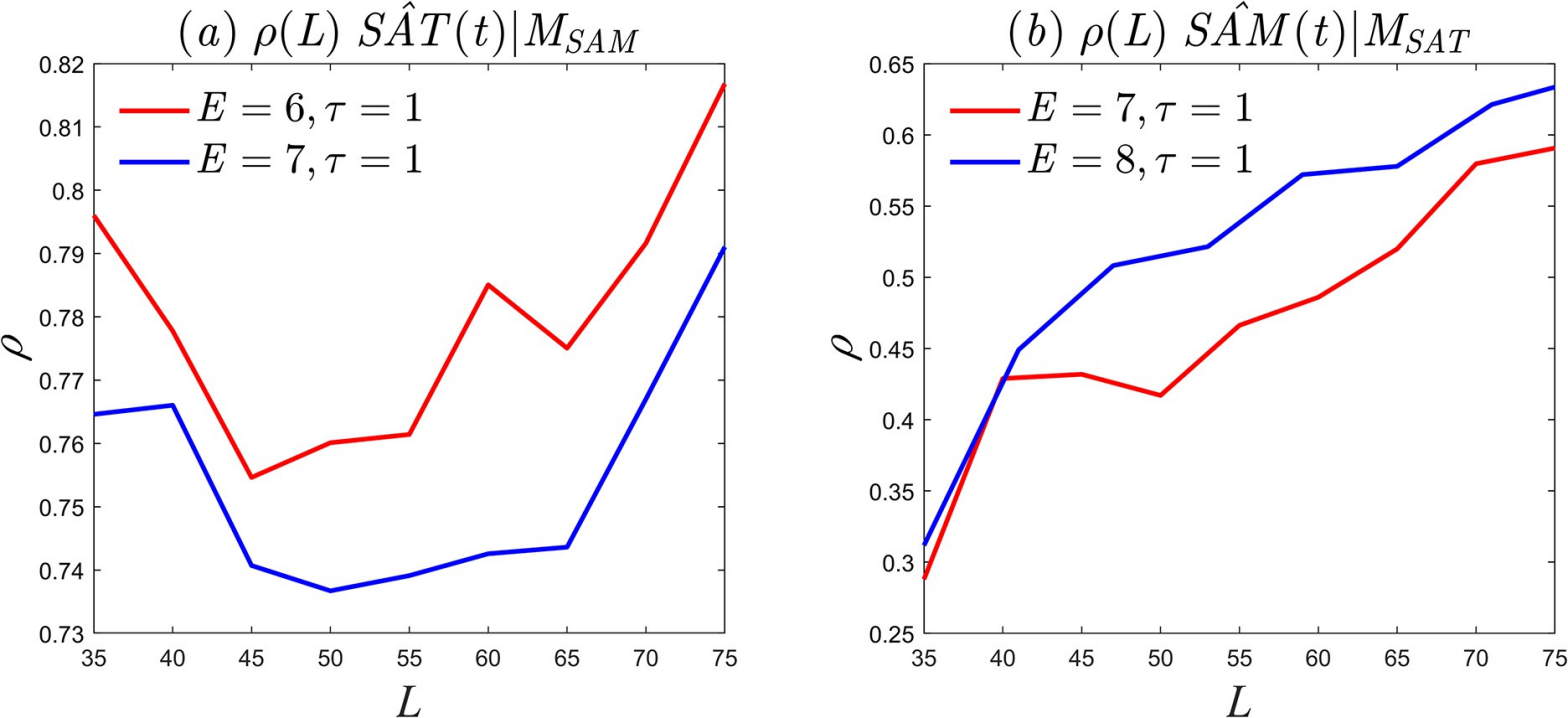
Causality curves. (a) Unidirectional causality of summer 1000*hPa SAT* in East Asia on summer *SAM*; (b) unidirectional causality of summer *SAM* on summer 1000*hPa SAT* in East Asia.

The parameter configurations represented by points *A* and *B* in Fig 7A were used to investigate the unidirectional driving force from the East Asian summer 1000*hPa SAT* to summer *SAM*. The results are shown in Fig 8A. As the length of the time series increases, the correlation coefficient does not converge to a stable value when *E* = 6 and *τ* = 1 (red solid line). In order to reduce the influence of randomness in the test results, the embedding dimension was increased to *E* = 7 and the time step was held at *τ* = 1, as shown by the blue solid line. As the length of the time series increases, the correlation coefficient curve still does not converge. It can be inferred that, under the available data, there is no unidirectional causality between East Asian summer 1000*hPa SAT* and summer *SAM*, but the predictive power cannot be dismissed. The prediction errors for both cases are small, only around 0.2, as shown in Fig 7A. Using the parameter configurations represented by points *A* and *B* in Fig 7B, the causality between summer *SAM* and East Asian summer 1000*hPa SAT* was investigated. Fig 8B shows that both causality curves exhibit a significant upward trend in the correlation coefficient as the length of the time series increases. With *E* = 7 and*τ* = 1, the prediction error is 1.31, as shown by the red solid line in Fig 8B. As the length of the time series increases, the correlation coefficient gradually converges to *ρ* = 0.65, suggesting that the strength of causality is *u* = 0.65. It can be inferred that summer *SAM* is the driving factor of 1000*hPa SAT* in the East Asian summer, with strong causality. With *E* = 8 and *τ* = 1, the summer *SAM* prediction error is 1.32, and the strength of causality is *ρ* = 0.63. The blue solid line in Fig 8B is maintained, which is completely consistent with Takens’ theory that a one-dimensional time series can always find an *E*-dimensional embedding phase space with unchanged topological meaning, satisfying *E*≥2*d*+1 (where *d* is the singular attractor dimension). When the embedding dimensions changes, the causality is not disrupted, but its strength changes.

**Fig 9:**
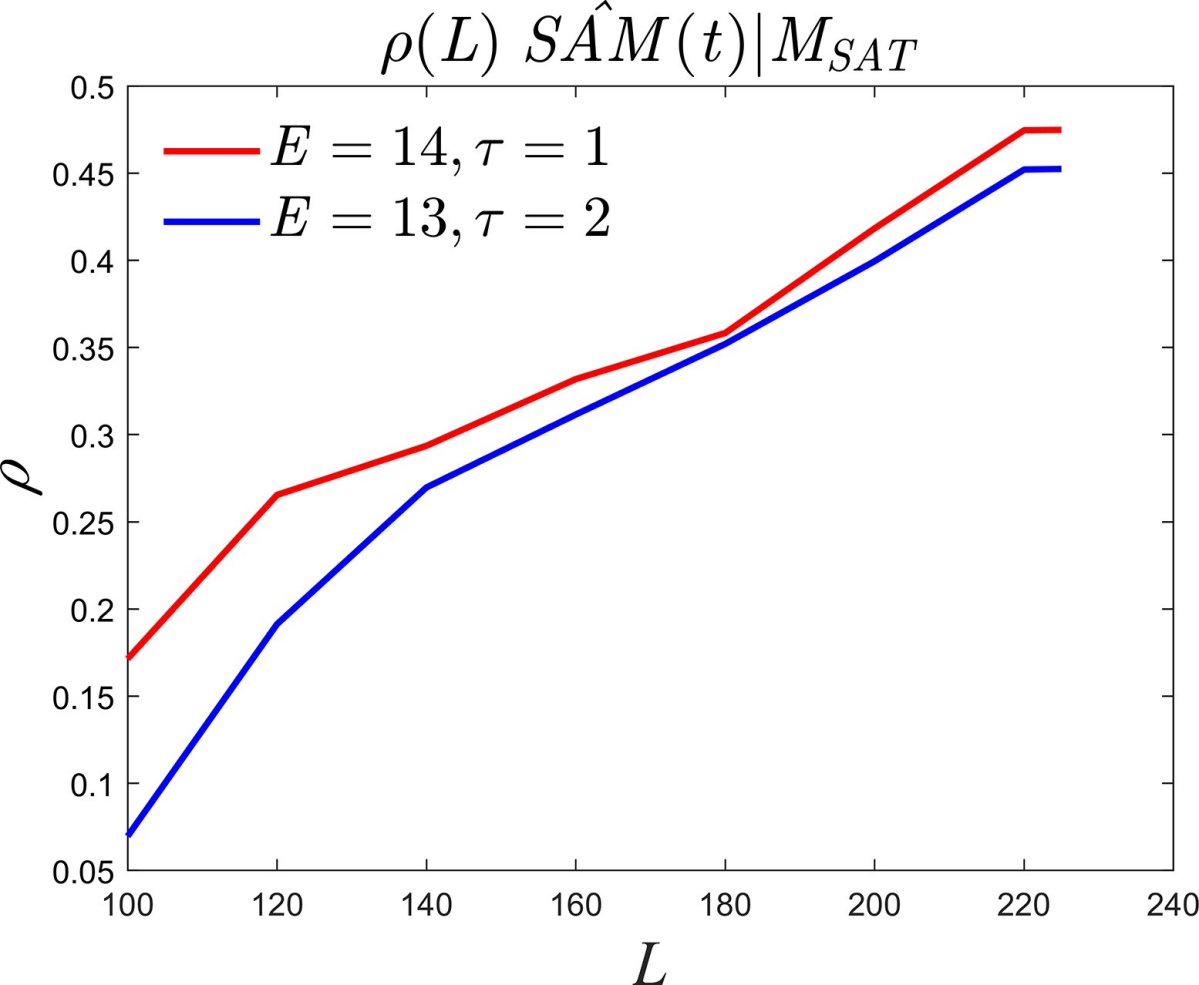
Monthly data causality curves.

We now examine the changes in causality as the density of observations increases. Monthly data were used to investigate the unidirectional causality between summer monthly *SAM* and summer 1000*hPa SAT* in East Asia. The optimal embedding dimension and time step parameter configurations and suboptimal parameter configurations are *E* = 14, *τ* = 1 and *E* = 13, *τ* = 2, respectively, as shown in Fig 9. Causality still exists, and the causal strength is about 0.47, which is slightly lower than the causal strength of 0.64 in Fig 8B.

In conclusion, our study confirms the existence of unidirectional causality between summer *SAM* and 1000*hPa SAT* in the East Asian summer. However, based on the available data, this causality is not strongly associated with data density, which contradicts the ideal test research findings. This may be the result of systematic errors (noise) in the observed data [38]. Furthermore, our results show that there is no increase in causal strength after encryption of the observed data.

### 4.3. Causality between summer SAM and EAMSI

**Fig 10:**
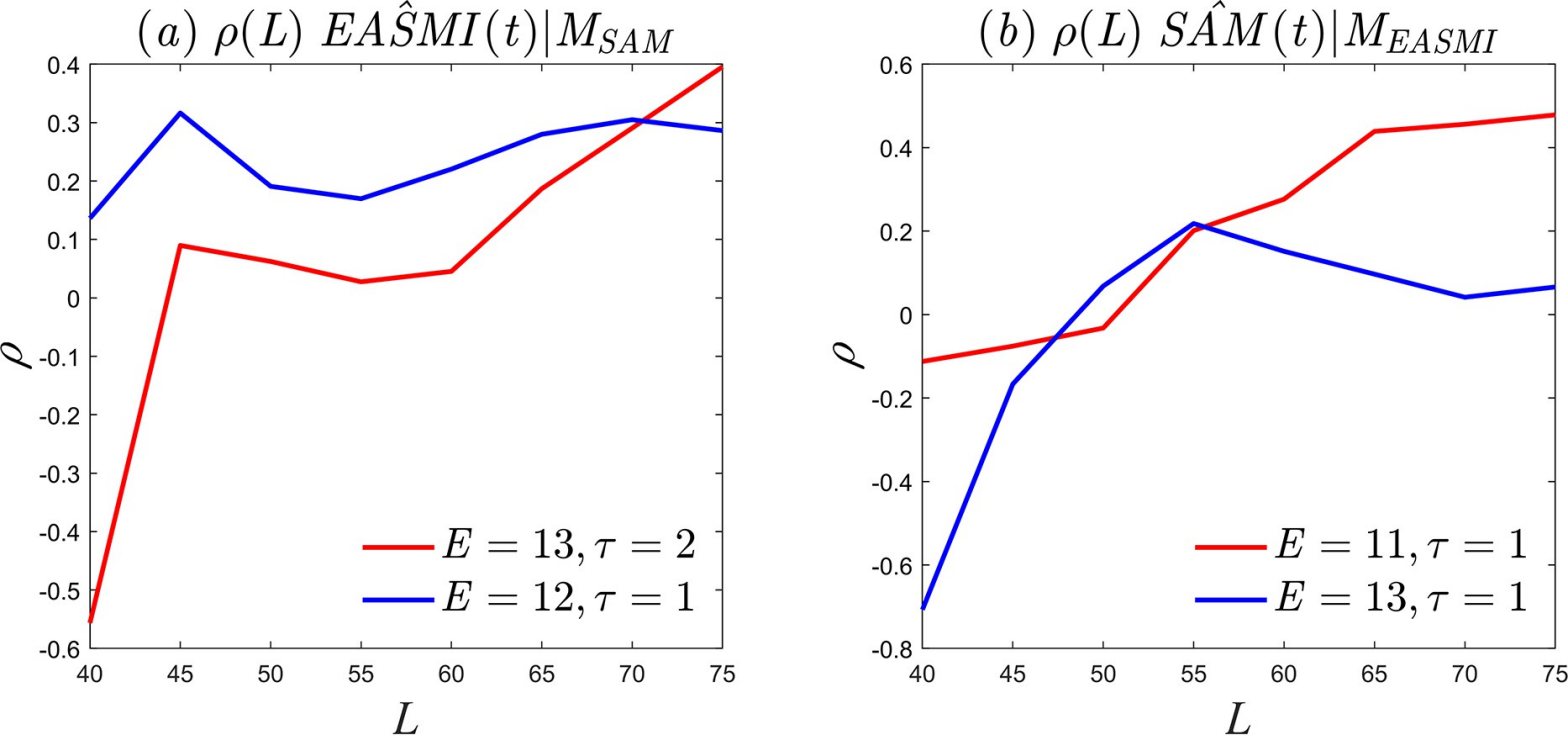
Causality curves. (a) Unidirectional causality of *EAMSI* on summer *SAM*; (b) unidirectional causality of summer *SAM* on *EAMSI*.

A similar discussion of causality between summer *SAM* and *EASMI* reveals that selecting the optimal parameter configuration as *E* = 13 and *τ* = 2 results in the failure of the unidirectional causality test of *EASMI* on summer *SAM* (indicated by the red solid line in Fig 10A). Even when selecting the second-best parameter configuration, *E* = 12 and *τ* = 1, the test still fails (indicated by the blue solid line in Fig 10A), which suggests the absence of unidirectional causality between *EASMI* and summer *SAM*. However, when testing the unidirectional causality between summer *SAM* and *EASMI*, the optimal parameter configuration of *E* = 11, *τ* = 1 and the second-best configuration of *E* = 13, *τ* = 1 both pass the significance test. Fig 9B shows that there is stable causality between *SAM* and *EASMI*, and the causal strength does not change significantly with changes in the embedding dimension. These results suggest the existence of unidirectional causality, where the summer *SAM* influences the motion trajectory of the *EASMI* dynamic system and serves as a reliable predictor. In summary, our research indicates that there is only one unidirectional causality between summer *SAM* and *EASMI*, which aligns with the physical mechanism of *SAM* propagating towards the northern hemisphere [37,39–42].

## 5. Conclusions and discussion

This paper has described the use of Takens’ theory and the CCM algorithm to detect causality in nonlinear climate systems. This analysis explains the intrinsic connections between elements of the climate system and explores the driving mechanisms of the atmospheric system. Through causality tests on both ideal models and atmospheric observation data, this study has demonstrated the feasibility of this detection method. The specific conclusions are as follows:

1. A method has been proposed for determining the optimal parameter configuration by selecting the optimal embedding dimension and time step in phase space reconstruction.
2. Causality between variables *X*, *Y*, and Z in the Lorenz equation was inferred, where *Y* and *Z* are the driving factors of *X*, *X* and *Z* are the driving factors of *Y*, with extremely strong causality. There also has a causality from *X* and *Y* to *Z*, although its strength is lower.
3. Using atmospheric observation data, the driving capability of *SAM* on *EASMI* and *SAT* was discussed. The results indicate that, during the summer season, *SAM* may affect *EASMI* and *SAT* through propagation across the equator. Specifically, *SAM* has a driving effect on both *EASMI* and *SAT* in East Asia, and this driving effect is unidirectional.

Causality detection indicates the existence of a causal driver, whereas failure to detect causality may be the result of limitations in existing data and methods, rather than the absence of causality. This is evident from the ideal experiment, where *X*↦*Z*,*Y*↦*Z* causality was found to exist but was not detected. Further research is needed to identify sufficient conditions for the absence of causal drivers, to evaluate the impact of time series observation errors on causality detection, and to detect causality in highly nonlinear systems.

## Supporting information

S1 File For observational data in this paper, please refer to the supplementary material. (XLSX)
